# CRISPR-enabled investigation of fitness costs associated with the E198A mutation in β-tubulin of *Colletotrichum siamense*

**DOI:** 10.3389/fpls.2023.1278133

**Published:** 2023-11-03

**Authors:** Scott D. Cosseboom, Chiti Agarwal, Mengjun Hu

**Affiliations:** Department of Plant Science and Landscape Architecture, University of Maryland, College Park, MD, United States

**Keywords:** CRISPR, *Colletotrichum*, fungicide resistance and management, fitness cost, E198A

## Abstract

**Introduction:**

Understanding fitness costs associated with fungicide resistance is critical to improve resistance management strategies. E198A in b-tubulin confers resistance to the fungicide thiophanate-methyl and has been widely reported in several plant pathogens including *Colletotrichum siamense*.

**Method:**

To better understand potential fitness costs associated with the resistance, a ribonucleoprotein (RNP) complex mediated CRISPR/Cas9 system was used to create a point mutation (E198A) through homology directed repair (HDR) in each of the sensitive (E198) *C. siamense* isolates collected from strawberries, raspberries, and peaches. The RNP complex was delivered into fungal protoplasts using polyethylene glycol-mediated (PEG) transfection.

**Results:**

The transformation efficiency, the proportion of transformants of sensitive parental isolates containing the E198A mutation, averaged 72%. No off-target mutations were observed when sequences similar to the b-tubulin target region with a maximum of four mismatch sites were analyzed, suggesting that the CRISPR/Cas9 system used in this study was highly specific for genome editing in *C. siamense*. Of the 41 comparisons of fitness between mutant and wild type isolates through in vitro and detached fruit assays, mutant isolates appeared to be as fit (24 of 41 comparisons), if not more fit than wild-type isolates (10 of 41 comparisons).

**Discussion:**

The use of CRISPR/Cas9 to evaluate fitness costs associated with point mutations in this study represents a novel and useful method, since wild-type and mutant isolates were genetically identical except for the target mutation.

## Introduction

*Colletotrichum* is one of the top 10 phytopathogenic fungi that is known to be associated with crown, leaf, and fruit diseases (Moreira et al., 2019). The diseases are typically referred to as anthracnose, but other names are also used such as bitter rot and ripe rot depending on crops. Among many species within the genus of *Colletotrichum*, *C. siamense* is one of the most important species causing pre- and post-harvest anthracnose (or different names) that reduce the yield and shelf-life of various fruits and vegetables, such as grapes, apples, blueberries, strawberries, and peppers (Dowling et al., 2020).

The methyl benzimidazole carbamates (MBC) are a widely used and effective group of fungicides due to their broad spectrum activity for disease control and potentially curative activity in the host plant (Olaya and Geddens, 2019). MBC fungicides work by inhibiting β-tubulin assembly during mitosis by binding to β-tubulin subunits in fungi (Davidse, 1986), and have been commonly used for anthracnose disease in fruit crops caused by the *C. gloeosporioides* complex including *C. siamense* (Hu et al., 2015; Luo et al., 2021). However, continuous exposure to MBCs have led to the development of fungicide resistance in *C. siamense* and other fungal populations (Ma and Michailides, 2005; Hu et al., 2015; Chung et al., 2020). With regard to the resistance mechanisms, point mutations at the 198- or 200-position codon in the β-tubulin (TUB2) gene have been associated with high levels of resistance to MBC fungicides (Ma and Michailides, 2005). Among them, E198A occurred the most frequently and conferred the highest level of resistance to MBCs in *Botrytis cinerea, Colletotrichum* spp., *Monilinia fructicola*, and *Trichoderma harzianum*, and only E198A has been reported in *C. siamense* (Chen et al., 2013; Li et al., 2013; Chung et al., 2020; Cosseboom and Hu, 2021).

Fitness costs associated with fungicide resistance has been investigated in some fungal pathogens (Chen et al., 2016). Previous studies have provided initial hints at fitness penalties, but these were often inconsistent (Hawkins and Fraaije, 2018). Traditional methods to study fitness costs primarily involved *in vitro* assays of isolates with different resistance phenotypes and genotypes (Chen et al., 2016). Because isolates used in individual studies typically had diverse genetic backgrounds, it has been impossible to exclude potential impacts on fitness from many genetic variations other than the specific mutation(s) conferring fungicide resistance. For instance, fungal isolates with resistance to MBCs showed normal growth and pathogenicity in different species, such as *Botrytis cinerea*, *Colletotrichum musae*, and *Monilinia fructicola* (Chen et al., 2014; Vieira et al., 2017; Hawkins and Fraaije, 2018). Yet, virulence was found to be even higher in *Penicillium expansum*, *Mycosphaerella fijiensis*, and *Didymella bryoniae* MBC-resistant isolates (Keinath and Zitter, 1998; Romero and Sutton, 1998; Baraldi et al., 2003). In contrast, the overall frequency of TUB2 sequences with E198A of any fungi submitted to GenBank has been in a decline since its peak between 2005 and 2009, following a similar, declining pattern of MBC fungicides usage between 1997 and 2003 in California and Arizona, USA (Bradshaw et al., 2021). This indicates that resistance to MBCs may generally entail a slight fitness disadvantage that previous *in vitro* studies have not been able to demonstrate. Yet, the decrease could also be due to diverse fungal species and variable number of TUB2 sequences submitted to GenBank each year.

The development of clustered regularly interspaced short palindromic repeats (CRISPR), associated with the RNA-guided Cas9 endonuclease CRISPR/Cas9 system, has significantly advanced gene editing in recent years (Nakamura et al., 2019; Yamada et al., 2023). Cas9 endonuclease and a single-guide RNA (sgRNA) are the two crucial CRISPR/Cas9 system components. The sgRNA sequence is about 20-nucleotides, which matches the designated locus for editing, which is flanked by a protospacer-adjacent motif (PAM) region unique to Cas9. This directs Cas9 to the editing site and results in an introduction of a double-stranded DNA break (DSB) (Jinek et al., 2012). For knock-in studies, these DSB are repaired by homology-directed repair (HDR) which require donor DNA homologous to the sequences flanking the DSB (Shalem et al., 2015). The delivery of the CRISPR/Cas9 system into a given organism’s genome can be made through a plasmid or plasmid-free approach such as a ribonucleoprotein (RNP). A RNP approach may be favored due to its reduced off-target effects and improved efficiency (Kim et al., 2014). Importantly, unlike plasmids, RNP-based transfections do not incorporate into the genome to form a “footprint” that could confound experimental results (Kim et al., 2014; Vakulskas and Behlke, 2019).

Replacements in targeted genes using CRISPR/Cas9 has been developed in a few *Colletotrichum* spp. and other filamentous fungi, but such a system has yet to be established for *C. siamense*. Additionally, no studies have been conducted to explicitly address fitness penalties associated with fungicide resistance using isolates with identical or largely the same genetic background. CRISPR/Cas9 can cause off-target alterations in the gene. Even though sgRNA sequences are chosen to avoid the occurrence of mutations in similar regions in the genome, off-site mutations have been observed in sites with several mismatches (Zhang et al., 2015). Thus, the main objectives of this study were to 1) produce the E198A mutation in TUB2 in *C. siamense* isolates using a plasmid-free RNP, 2) confirm no off-target mutations, and 3) investigate fitness and competitive ability of mutants (MT) with the E198A compared to wild-type (WT) strains. Understanding the fitness components of resistant populations will serve as the foundation for determinations of whether a fungicide should be reintroduced into a field where resistance has been documented, or for refining other resistance management tactics (Ma and Michailides, 2005).

## Materials and methods

### Design of crRNA, ssODN, and *in vitro* assembly of a Cas9/sgRNA ribonucleoprotein complex

For CRISPR/Cas9 ribonucleoprotein (RNP) complex-mediated gene editing, Cas9-sgRNA constructs were employed. β-tubulin (TUB2) sequence data was obtained from a WT (E198) *Colletotrichum gloeosporioides* isolate from GenBank (accession U14138). To produce the E198A mutation from thiophanate-methyl sensitive isolates, the glutamic acid (E) encoding sequence “GAG” was changed to alanine (A) coding sequence “GCG” at codon 198 of TUB2. The CRISPR/Cas9 guide RNA Design Checker (Integrated DNA Technologies Inc. IDT, Coralville, IA) was used to design CRISPR-RNAs (crRNAs) by screening the target site of the β-tubulin gene using “NGG” as the protospacer adjacent motif (PAM). The best scoring crRNA sequence was checked using BLAST analysis on the *C. siamense* reference genome (GenBank assembly ASM1339019v1) to avoid potential off-target activity. The crRNA (5’- GUCAAUGCAGAAGGUCUCGU-3’) and tracrRNA (5’-GUUUUAGAGCUAUGCU-3’) sequences were ordered as oligonucleotides from IDT. 100 μM stock solutions of lyophilized crRNA and tracrRNA were prepared using nuclease-free duplex buffer, supplied by IDT, and stored at -20°C until use. The Cas9 enzyme was delivered from IDT at a concentration of 100 µg/µl and stored at -20°C until use.

**Figure 1:**
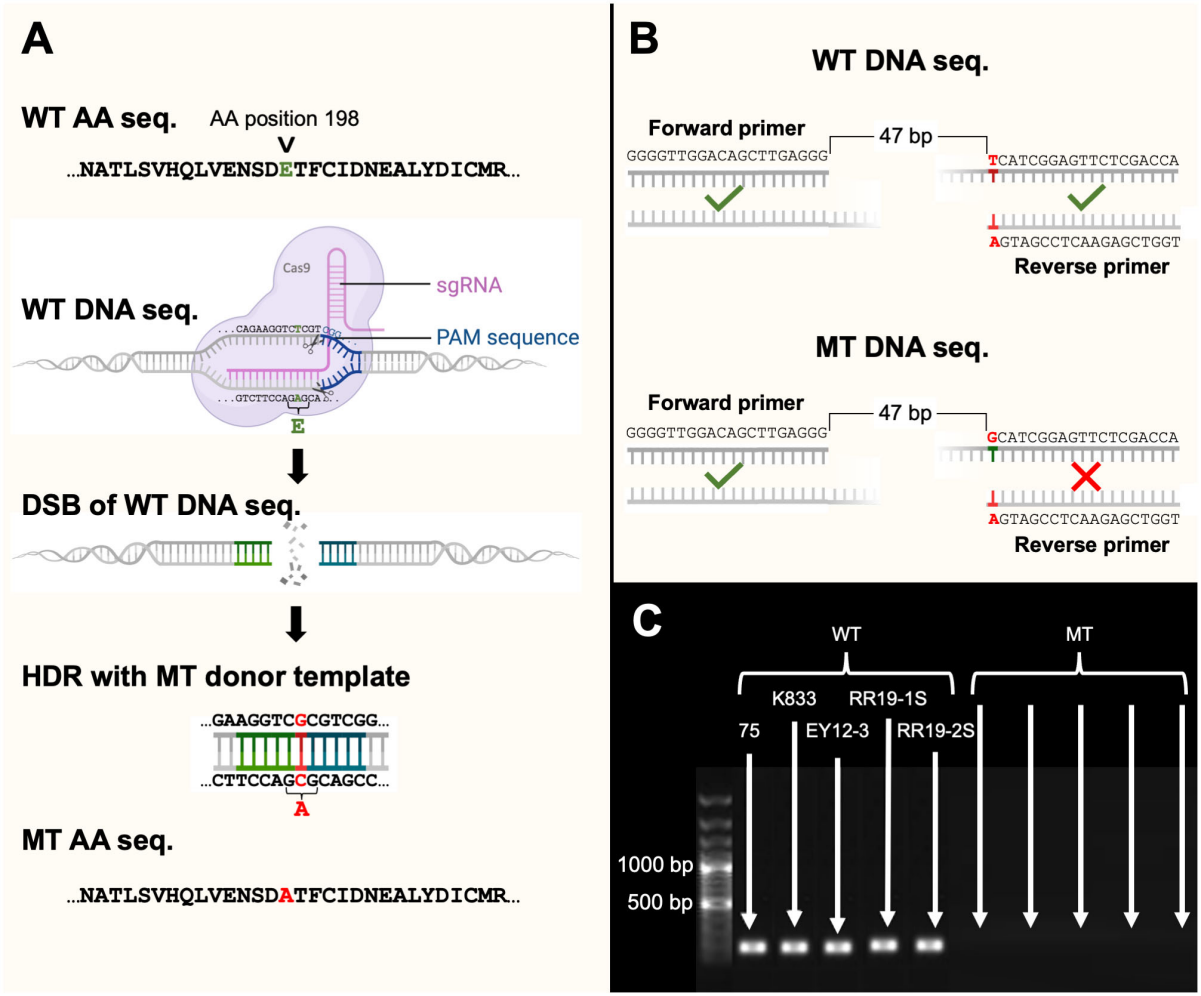
**(A)** An illustration of a ribonucleoprotein complex consisting of single-guide RNA (sgRNA) and Cas9 endonuclease which mediated the transformation of wild-type (WT) TUB2 genotype to a mutant type (MT) genotype by causing a double stranded break (DSB) followed by homology directed repair (HDR), **(B)** an illustration of an allele-specific PCR assay designed to only amplify WT DNA and not MT DNA, and **(C)** a 1% agarose gel consisting of a 100 bp marker, and DNA of five WT and five MT isolates amplified with the allele-specific primers. This figure was created with BioRender.com.

To prepare the two-part sgRNA complex, the crRNA was hybridized to equal molar concentrations with tracrRNA in nuclease-free duplex buffer (pH 7.5) at a concentration of 50 µM. The mixture was annealed by heating at 95°C for 5 min and then slowly cooling down to room temperature (24°C). The resulting sgRNA was used immediately. To generate the Cas9 ribonucleoprotein complex, 3 µl of sgRNA solution (150 pmol), and 2 µl of Cas9 enzyme solution (125 pmol) (1 µg/µl in the IDT reaction buffer) were mixed. The mixture was incubated at room temperature for 20 min to allow ribonucleoprotein complex formation. A single-stranded donor oligonucleotide (ssODN; 5’-GCCACTCTCTCCGTCCACCAGCTGGTCGAGAACTCCGACGCGACCTTCTGCATTGACAACGAGGCTCTCTACGACATTTGC-3’) for the HDR was designed with homology arms in both orientations (5’-3’;3’-5’), complementary to the target DNA strand. The ssODN sequence included the desired mutated nucleotide between the homology arms (**Figure 1A**). The ssODNs were synthesized as ultramer oligonucleotides by IDT.

### Protoplast preparation

Five *C. siamense* isolates previously collected and identified from different small fruit hosts, including two from strawberry (75 and K833), one from peach (EY12-3), and two from red raspberry (RR19-1S and RR19-2S) were used in this study (Hu et al., 2015; Schoeneberg and Hu, 2020; Luo et al., 2021). These isolates were previously found to be sensitive to thiophanate-methyl with a WT genotype (E198). The isolates were revived from filter paper stocks on potato dextrose agar (PDA). Dense suspensions of mycelia of each isolate were obtained by inoculating actively growing mycelial plugs (2 mm in diameter) into 100 ml potato dextrose broth (PDB) (Becton Dickinson, Sparks, USA), followed by incubation at 27°C for five days.

For polyethylene glycol (PEG)-mediated transformation of protoplasts, a previously developed method (Redman and Rodriguez, 1994) was modified and utilized in this study. A confluent mass (approx. 3 grams) of mycelia was harvested after 5 to 7 days when it covered the surface of the PDB. The mycelia were washed with sterile water and protoplast buffer (0.8 M MgSO4, 0.2 M sodium citrate, pH 5.5) over a 250 ml funnel with 4 layers of sterile cheesecloth. Washed mycelia were coarsely chopped and digested with 300 mg Glucanex enzyme in 3 ml of Novozyme buffer (1 M sorbitol, 50 mM sodium citrate, pH 5.8) and filtered with a 0.45 µm syringe filter, followed by the addition of 17 ml protoplast buffer and incubation at 27 °C for 5 hours. Residual mycelia were then separated from protoplasts by filtration through glass wool, and 30 ml of KC1 (0.6 M) was added to the filtrate. Protoplasts were pelleted in conical tubes at 5000 RPM at 4°C. The resulting pellets were washed with 15 ml STC buffer (1M sorbitol, 50mM TRIS, pH 8, 50 mM CaCl2.2H2O), recentrifuged 5000 RPM at 4°C and suspended in STC to a final concentration of 2x10^8^ protoplasts per ml.

### Protoplast-mediated transformation of *C. siamense*

Protoplasts (20 µl) were mixed with 5 µl of the RNP complex, 1.2 µl of ssODN solution (100 µM Alt-R-IDT), and 0.1 mM ethylenediamine­tetraacetic acid and incubated on ice for 20 min in 15-ml polyallomer tubes. Next, the isolated protoplasts were mixed with 200 µl of PEG solution (60% polyethylene glycol 3800, 10 mM Tris-HCI, pH 7.5, and 100 mM CaCl_2_) and incubated at room temperature for 10 min. Finally, the protoplast suspension was diluted to a final volume of 5 ml with a regeneration medium (0.6 M mannitol, 0.1% casamino acids, and 0.1% yeast extract, pH 5.3). The tubes were placed horizontally for 24 h under white fluorescent lights at 22°C, and 100 µl of the regeneration suspension was plated onto HM media plates (13.85% mannitol, 0.1% casamino acids, 0.1% yeast extract, 0.4% sucrose, and 2.0% agar) in the presence or absence of thiophanate-methyl (Topsin M WSB, United Phosphorous Inc., King of Prussia, PA) at 100 µg/ml. Plates were incubated at 22°C under white fluorescent lights (Redman and Rodriguez, 1994). After seven days of incubation, emerging fungal colonies, considered transformants, were transferred to fresh PDA plates.

### Verification of E198A mutants and efficiency of CRISPR/Cas9

Mycelia of transformants from the fungicide amended HM plates were used for genomic DNA extraction. Allele-specific primers (5’- TGGTCGAGAACTCCGATGA-3’ and 5’- GGGGTTGGACAGCTTGAGGG-3’) were designed to amplify the target site to confirm the absence of the nucleotide ‘A’ in resistant mutants (**Figure 1B**). Furthermore, 11 randomly selected mutants were amplified using primers TARGETF/TARGETR (**Table 1**) designed for gRNA and were Sanger sequenced. The PCR amplification was conducted with a total volume of 25 µl containing 2X master mix (Apex Bioresearch Products, San Diego, CA), 0.25 µM of each primer, 1 µl of DNA, and pure water. The thermocycling conditions were 94°C for 3 minutes followed by 35 cycles of 94°C for 40 seconds, 55°C for 40 seconds, and 72°C for 2 minutes, and then a final step of 72°C for 5 minutes. Amplicons were purified with the Exosap-IT reagent (Applied Biosystems, Waltham, MA) and were Sanger sequenced by Genewiz (www.genewiz.com). The sequences were analyzed using Snapgene software (Insightful Science; available at snapgene.com). Mycelia of transformants from the non-fungicide-amended HM plates were transferred to the 100 µg/ml thiophanate methyl amended PDA plates to determine the efficiency of the RNP complex. The efficiency was the percentage of transformant isolates which were able to grow on the amended media. The stability of the E198A mutation in *C. siamense* transformants was assessed by four consecutive transfers of each transformant onto fungicide non-amended PDA plates and then to a thiophanate-methyl amended PDA plate at 100 µg/ml.

**Table 1:** Primers used to amplify regions identified by the Cas-OFFinder tool with sequences that matched or nearly matched the crRNA sequence.

Primer Name	Sequence (5’ to 3’)	No. Mismatches
OFFTARGETF-1	CCTTTTCTTGCCTACCCGCT	4
OFFTARGETR-1	GTGGACTTCCCAGTCGCTTG	
OFFTARGETF-2	GCTATTTCTGGCACCAACCG	4
OFFTARGETR-2	TTATGGGCGGGTAGTGTGTG	
OFFTARGETF-3A	TGCCATAGGCGTCAAGAGAC	4
OFFTARGETR-3A	GACAAGCGAACTATGCCTGC	
OFFTARGETF-3B	GTCAAGAGACCTGCTCCACT	4
OFFTARGETR-3B	GGTTTTCCAACGAGAAGGCAG	
OFFTARGETF-4	GCTTGTGTGACTCGGCTAGA	4
OFFTARGETR-4	CGTCAGGTCGTTCGTTACCT	
OFFTARGETF-5	GGAGCCAGATGTGGGAACTC	4
OFFTARGETR-5	TTCGTCCCTTGAGGCTGTTC	
TARGETF	GGGTACGCATGCAAATGTCG	0
TARGETR	CCCTACAACGCCACTCTCTC	

The effective concentration of thiophanate-methyl that limited growth by 50% (EC_50_) of MT and WT strains was evaluated with a 96-well SmartReader96 photometer (Accuris USA, Edison, NJ) as described previously (Cosseboom and Hu, 2022). Each well of the 96-well flat-bottom plates contained 100 µl of PDB, 10,000 conidia, and either 0, 0.01, 0.1, 0.3, 1, 3, 10, or 100 µg/ml thiophanate-methyl. The plates were incubated in a plastic box at 28°C for 24 hours before measuring the growth with the 405 nm filter on the photometer.

### Off-target mutation search

The program Cas-OFFinder (Bae et al., 2014) (http://www.rgenome.net/cas-offinder) was used to check for off-target matches of the sgRNA where potential off-target gene editing could have occurred. This tool checked for locations on the *C. siamense* genome that matched the crRNA sequence and allowed for a specified number of mismatches. The higher the number of mismatches, the less likely off-target editing would occur. The mismatch number was set at four or less, and the tool revealed the locations of these sequence matches in the genome. Primers were designed to amplify the sequence of each of these regions (**Table 1**). A PCR was performed using the primers for each mismatch sequence obtained from the Cas-OFFinder tool with 11 resistant transformants to check for possible off-target mutations. The PCR was conducted as described above, and amplicons were Sanger sequenced and analyzed for the sequence “GCG” instead of “GAG” within the crRNA-matching region.

### Multi-temperature fitness assessments

Then, the effect of different temperatures on mycelial growth rate, sporulation, and spore production of WT and their respective MT isolates was evaluated to determine the fitness costs associated with resistance. Mycelial plugs (5 mm diameter) of the five WT isolates and three MT isolates from each of the five parental strains were transferred from 5-day-old cultures to the center of PDA medium in 90 mm petri dishes. A 5 mm cork-borer was used to transfer one mycelial plug placed face down onto each PDA plate. Three plates of each isolate were incubated in complete darkness at 16, 24, and 32°C for 4 days. From each isolate’s colony, radial measurements of mycelial growth were measured with a digital caliper.

The same isolates were plated on ¼ strength PDA and incubated at the same three temperatures for 7 days to induce sporulation. Spores that formed on each plate were submerged in 8 ml of a 1% Tween 20 solution and suspended with a sterile cell spreader and filtered through triple-layered cheesecloth. The conidial concentration of the suspension was measured using a hemocytometer. This concentration was multiplied by the volume in which each isolate was rinsed (8 ml) to calculate the total spores produced on each plate. Spore suspensions from 3 plates per isolate were counted as replicates.

A spore suspension from each isolate counted above were diluted to 1×10^5^ spores/ml to be used in the spore germination assay. Next, 50 µl of conidial suspension was transferred to petri plates containing PDA and a cell spreader was used to spread the suspension evenly across each plate. Plates were incubated for 4 and 8 hours at 16, 24, and 32°C in continuous darkness. 30 conidia were examined at three regions of each plate with a compound microscope (40x) to determine the germination percentage. If the germ tube of conidia was at least half the spore length, it was considered germinated. Each of these experiments were conducted twice.

### Multigenerational fitness assays

As previous research has found changes in behavior of fungi that have been sub-cultured multiple times (Breen et al., 2022), fitness metrics were conducted at a fixed temperature of 28°C and under constant fluorescent light, but were tested over multiple generations. One WT and MT isolate of each strain and three total strains were revived from frozen stocks and were plated on ¼ strength PDA and were regarded as generation 0. Once the colonies sporulated, spores were transferred to fresh ¼ strength PDA plates and regarded as generation 1. This process was continued up until generation 5. The same spore suspensions were diluted to 4x10^5^ conidia/ml and were used to inoculate plates and fruit for the subsequent fitness assays.

15 µl of the spore suspensions was pipetted onto the center of PDA plates for the mycelial growth rate assays, which were conducted as described above. The same amount of spore suspension was pipetted onto the center of three ¼ strength PDA plates per isolate for the sporulation rate assays which were then conducted as described above for the sporulation rate assessment. The spore suspensions were also spread on PDA plates as described above for the germination rate. The same spore suspensions were utilized to evaluate the sensitivity (EC_50_) to two fungicides not related to MBCs, difenoconazole and penthiopyrad. These two fungicides belonged to different chemical classes, the demethylation inhibitors (DMIs) and the succinate dehydrogenase inhibitors (SDHIs), respectively. This was conducted with the same photometer settings and concentrations as described above. Each of these assays were conducted twice.

### Detached fruit assays

Spore suspensions (1x10^5^ conidia/ml) of WT and MT isolates were utilized to inoculate store-bought, organic blueberry and apple fruit. The fruit were surface sterilized in 1% sodium hypochlorite for one minute, rinsed with sterile water twice, and dried. Each apple fruit was wounded twice by inserting a sterile toothpick to a depth of 5 mm at two points on the surface of the side of the fruit at a 30 mm distance, and then 15 µl of the 1x10^5^ conidia/ml spore suspensions were pipetted into the wound. The WT spore suspension was inoculated into one of the wounds and its respective MT spore suspension was inoculated into the other wound on the same fruit for eight fruit per WT/MT combination (three strains were included). The fruit were placed on 60 mm petri dish lids within plastic boxes. The bottoms of the boxes were flooded with sterile water to maintain humidity. The boxes were incubated at 28°C for seven days, and the diameter of lesions on the apples was measured with a digital caliper.

Blueberries were divided into groups of 20 and evenly spaced on wire mesh within water-tight food takeout containers. 4.5 ml of each spore suspension was misted onto blueberries in four of the containers, which were then covered with the lid to maintain moisture and incubated at 28°C. The blueberry disease incidence was measured at 3-, 4-, and 5-days post-inoculation by counting the number of fruit displaying anthracnose symptoms and orange sporulation. The blueberry incidence data was transformed into area under the disease progress curve (AUDPC) values for analysis. Each of these experiments were conducted twice.

### Multigenerational competition assays

WT and MT strains were evaluated for their competitivity by inoculating blueberries with equal parts of spore suspensions of WT and MT spores. Conidia of WT and MT isolates of three strains (75 MT/WT, K833 MT/WT, and EY12-3 MT/10WT) were suspended in sterile deionized water and the suspensions were diluted to 1x10^5^ conidia/ml. 2.5 ml of each WT suspension was combined with 2.5 ml of their respective MT suspensions of the same strains (generation 0). The blueberries were misted with this spore suspension mixture and incubated until symptom development as described above. 0.5 ml of this and all subsequent spore suspensions were stored at -20°C. Once symptoms, namely visual orange sporulation on the surfaces of the berries, were observed, conidia were washed off the berries, resulting in spore suspensions that were again diluted to 1x10^5^ conidia/ml (generation 1). These suspensions were used to inoculate fresh blueberries as described above. This was repeated for four generations. This was conducted twice for each WT/MT strain combination. After the detached fruit experiments were completed, each 0.5 ml spore suspension was thawed and centrifuged at 12,000 rpm for 6 minutes. 0.3 ml supernatant was discarded, and DNA was extracted from the 0.2 ml remaining volume with the Omega HP Fungal DNA extraction kit (Omega Bio-Tek Inc., Norcross, GA). The resultant DNA was evaluated for the quantity of MT and WT DNA with TaqMan qPCR.

Primers (5’-TCCAAGATCCGTGAGGAGTTC-3’ and 5’- CACCGGACATAACAGCAGAGA-3’) targeting a 250 bp region of the β-tubulin gene in the *C. siamense* genome were designed for amplification in qPCR based on the TUB2 sequence of one of the parental isolates. Probes that were specific to either the WT (5’- AGAATTCCGACGAGACCTTCTGCATT-3’) or the MT (5’- AGAATTCCGACGCGACCTTCTGCATT-3’) sequence were designed and were labeled with the black hole quencher (BHQ-1) on the 3’ end and FAM or HEX on the 5’ end, respectively. Each qPCR reaction consisted of 20 µl total volume, with TaqMan Fast Advanced Master Mix (Applied Biosystems, Waltham, MA), 0.3 µM of each probe, 0.2 µM of each primer, 3 µl of DNA, and DNA-free water. The thermocycling conditions were 95°C for 2 min, then 45 cycles of 95°C for 15 seconds followed by 60.7°C for 45 seconds. A standard curve ranging from 1 ng/µl to 0.001 ng/µl of WT and MT DNA validated the sensitivity and efficiency of the assay (**Supplementary Figure 1**). Also, DNA was extracted from spore suspensions containing known ratios of WT : MT spores: 0:100, 25:75, 50:50, 75:25, 100:0, and 0:0 and was amplified in qPCR. This was also conducted with the DNA extracted from the multigenerational blueberry experiments with each qPCR plate containing the six known ratios. Each sample was included in triplicate wells in each plate. The ΔC_t_ method was used to calculate the ratio of WT to MT DNA from the qPCR results (Germer et al., 2000).

### Statistical analysis

Statistical analysis was performed individually for each fitness component using JMP Pro Version 15 software (SAS Institute, Cary, NC). A Levene’s test was conducted to determine if variances were similar between the two independently repeated experiments before combining the data for comparisons. The mycelial growth rate values, apple lesion diameters, blueberry incidence AUDPC values, and penthiopyrad and difenoconazole EC_50_ values were compared with a generalized linear model including the isolate type (MT or WT) and incubation temperature treatments (if multiple temperatures were included) as a fixed effect and the experiment and “strain” as a random effect. If the fixed effect was significant, then a *post hoc* T-test was conducted. For the spore production and germination rate experiments, the residuals were not normally distributed, so a Wilcoxon test was used to evaluate the difference between the isolate type-temperature effect. The multigenerational fitness assay data was analyzed similarly, but the generation variable was also included as a fixed effect. A repeated measures analysis was conducted on the frequency of WT to MT DNA from the qPCR competition assays to determine if the generation influenced the frequency. In this analysis, the generation was a fixed effect and the strain and replicate were random effects.

## Results

### Validation of E198A in the *C. siamense* mutants

The CRISPR/Cas9 mediated point mutation of E198A in the TUB2 gene was successfully accomplished by the transient introduction of purified Cas9 protein and *in vitro* synthesized sgRNAs into protoplasts of five *C. siamense* isolates. Cas9-NLS-sgRNA ribonucleoprotein complexes (RNPs) were introduced into all five sensitive parental isolates, together with donor DNAs which introduced an insertion containing the desired base for the E198A point mutation by repair of the DSB by homologous recombination with donor DNAs containing homologous regions on either side of the desired base (**Figure 1A**). After the protoplasts had undergone transformation, HM-containing thiophanate-methyl (at a concentration at which only resistant isolates could grow) selected for successfully mutated isolates. The sensitivity (EC_50_) of the WT and MT isolates was evaluated, and the WT isolates averaged 3.6 µg/ml while the MT isolates were all greater than 100 µg/ml. The TUB2 genotype of each transformant isolate was characterized with allele-specific primers in PCR that only amplified the sensitive, parental genotype (E198) and not the resistant, mutant genotype (E198A; **Figures 1B, C**). This characterization was further confirmed by sequencing the TUB2 gene of 11 single-spored mutant isolates. Sequences matched the allele-specific PCR, with resistant isolates revealing successful replacement of E with A at codon 198.

Mycelia from each resistant transformant were transferred from HM plates to non-amended PDA plates four consecutive times followed by plating on thiophanate-methyl amended PDA for the stability test. Each transformant was able to grow on the fungicide amended PDA plates, demonstrating stable resistance.

### The efficiency and accuracy of CRISPR/Cas9 system gene-targeting in *C. siamense*

Homology-dependent repair (HDR) mediated gene targeting efficiency was checked separately for all resistant mutants generated from each of the five *C. siamense* parental isolates. The point mutation made the isolates thiophanate-methyl resistant, which enabled distinguishing resistant mutants from sensitive parental isolates by their ability to grow on the fungicide amended plates. Efficiency ranged from 60 to 80% between the five isolates, with 80% efficiency from both strawberry isolates, 70% and 60% from the two raspberry isolates, and 70% from the peach isolate (**Table 2**).

**Table 2:** The efficiency rate of CRISPR/Cas9 using an RNP complex in *C. siamense* isolates collected from different hosts.

Isolate name	Host	Efficiency
K833	Strawberry	80%
75	Strawberry	80%
RR19-1S	Raspberry	70%
RR19-2S	Raspberry	60%
EY12-3	Peach	70%

Five regions with similar sequences to the gRNA target site with up to four mismatched bases were Sanger sequenced, and no mutations were observed in any of the mutants in these off-target regions. This was conducted for 12 isolates and the target region was also sequenced. The target region of the mutants had glycine in the place of alanine in position 198, while the off-target regions only had alanine.

### *In vitro* fitness assessments

**Figure 2:**
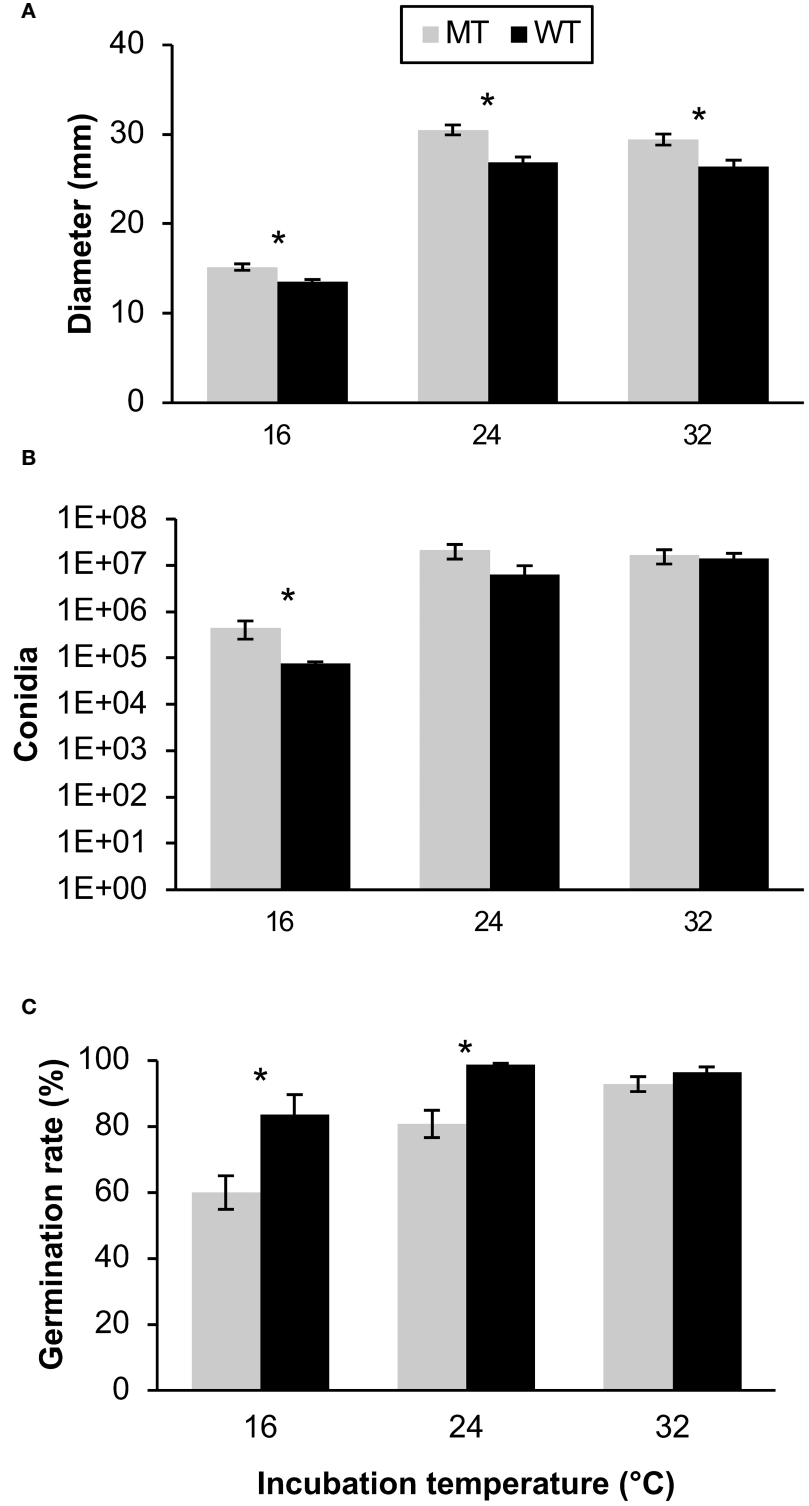
Multi-temperature fitness assessments of 5 wild-type (WT) and 15 mutant (MT) *Colletotrichum siamense* isolates respectively containing E198 and E198A in the TUB2 gene including **(A)** the colony diameters after 4 days incubation, **(B)** the quantity of spores produced after 7 days incubation, and **(C)** the percentage of germinated spores observed after 8 h incubation. Pairs of columns with an asterisk (*) are significantly different according to a T-test or a Wilcoxon test (α = 0.05) and error bars are standard error of the mean.

**Figure 3:**
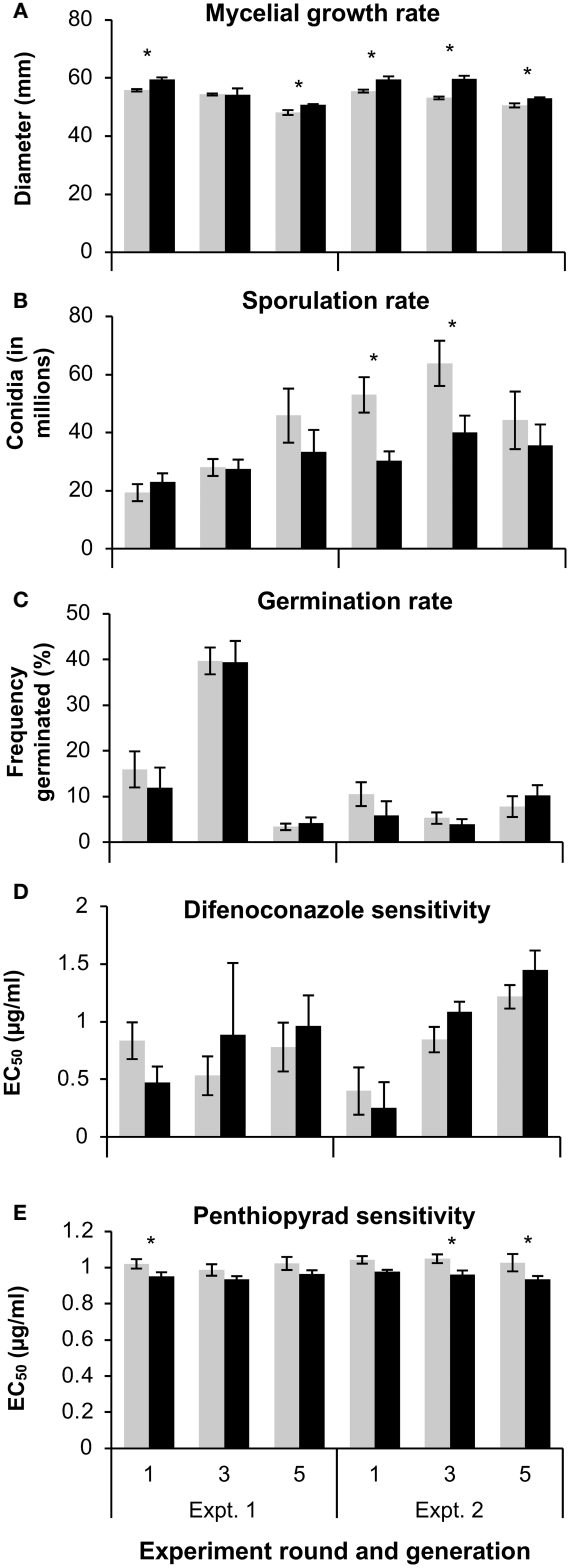
The **(A)** mycelial growth rate, **(B)** sporulation rate, **(C)** germination rate, and the effective concentration of **(D)** difenoconazole or penthiopyrad **(E)** that limited growth by 50% (EC_50_) of 3 wild-type (WT) and 3 mutant (MT) *Colletotrichum siamense* isolates that were successively replated for up to five generations on artificial media. Pairs of columns with an asterisk (*) are significantly different according to a T-test or a Wilcoxon test (α = 0.05) and error bars are standard error of the mean.

The fitness of fungicide-resistant mutants and sensitive *C. siamense* isolates were assessed by measuring mycelial growth, conidial production, and conidial germination rate on fungicide-free media at multiple temperatures. Also, the mycelial growth rate, sporulation rate, germination rate, difenoconazole sensitivity, and penthiopyrad sensitivity were evaluated at the first, third, and fifth generation of WT and MT isolates after reviving from storage. The variance between the two repeated assays were not equivalent for some of the multigenerational fitness tests, so each repetition of these experiments was evaluated individually. A Levene’s test confirmed that the variation in the data from the two independent multi-temperature experiments was equivalent for the mycelial growth (p = 0.104), spore production (p = 0.272), and germination rate assays (p = 0.094), so the data from the two experiments was combined for analysis. In the multi-temperature mycelial growth assays, MT isolates grew significantly larger than the WT isolates at each temperature (p < 0.001). Overall, the colony sizes were larger at 24 and 32°C than at 16°C (**Figure 2A**). Contrastingly, the WT isolates grew significantly larger than MT isolates at generation 1 and 5 during the first experiment and at generations 1, 3, and 5 of the second experiment (**Figure 3A**).

The MT isolates produced significantly more spores than the WT isolates at the 16°C incubation temperature (p < 0.031), and no differences between the isolate types were observed at the 24 and 32°C temperatures (**Figure 2B**). The 24 and 32°C treatments also produced more spores than the 16°C temperature treatment. The variation in sporulation rate between MT and WT isolates was larger in the second multigenerational experiment and the MT produced more spores than the WT in generations 1 and 3 in this experiment (**Figure 3B**). Overall, MT isolates produced an average of 42.4x10^6^ conidia, while WT isolates produced an average of 31.4x10^6^ conidia.

In the multi-temperature spore germination experiments, the isolate type had a significant effect on the germination rate. The germination rate was measured after 4 and 8 hours. Between the repeated experiments the germination rate at the 4-hour time point was highly variable, and therefore was not statistically analyzed. At the 8-hour point, the WT isolates had a significantly higher germination rate than the MT isolates at the 16 (p = 0.024) and 24°C (p = 0.010) temperature treatments (**Figure 2C**). In the multi-generational germination rate assays at 28°C, almost all spores germinated after eight hours of incubation (data not shown), but more variation was observed after four hours of incubation. There were no significant differences in germination rate between MT and WT isolates in any generation (**Figure 3C**).

In the multigenerational fungicide sensitivity assays, there were no significant differences between MT and WT isolates in any generation for the difenoconazole sensitivity assays (**Figure 3D**). Overall, the average EC_50_ value to difenoconazole was 0.82 and 1.05 µg/ml for MT and WT isolates, respectively. With regards to penthiopyrad sensitivity, MT isolates had a significantly higher EC_50_ value than WT isolates at generation 1 of the first experiment and generation 1 and 5 of the second experiment (**Figure 3E**). Overall, the average EC_50_ value to penthiopyrad was 1.03 and 0.95 µg/ml for MT and WT isolates, respectively.

### Detached fruit assays

**Figure 4:**
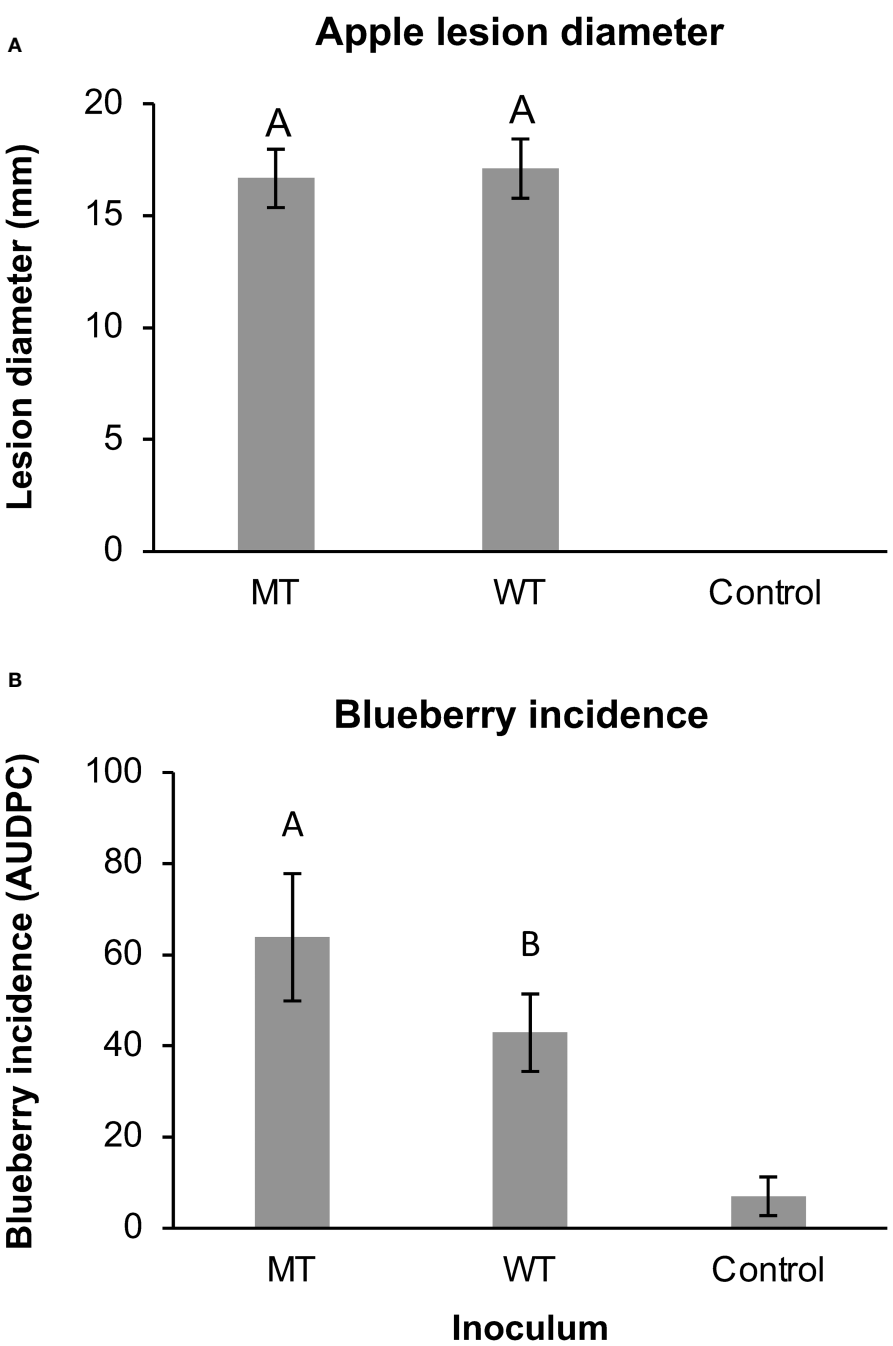
The **(A)** lesion diameters of wounded apples and **(B)** the incidence of anthracnose on non-wounded blueberries inoculated with 3 wild-type (WT) or 3 mutant (MT) isolates of *Colletotrichum siamense* after incubation at 28°C for 7 and 5 days, respectively. Columns with different letters are significantly different and error bars are standard error of the mean. The control treatments were not included in the statistical analysis.

In the detached apple and blueberry assays, the diseases caused by *C. siamense* appeared similar to previously observed symptoms of apple bitter rot and blueberry ripe rot (Verma et al., 2007). In the apple assays, there were no significant differences between the WT and MT lesions diameters (p = 0.696; **Figure 4A**). The blueberry incidence averaged 39.2% with MT-inoculated berries and 35.4 with WT-inoculated berries, and the respective MT AUDPC values were significantly higher than the WT AUDPC values (p = 0.0146; **Figure 4B**). Strain did not have a significant effect on the blueberry incidence (p = 0.322) or apple lesion diameter (p = 0.323).

### Competition assays

**Figure 5:**
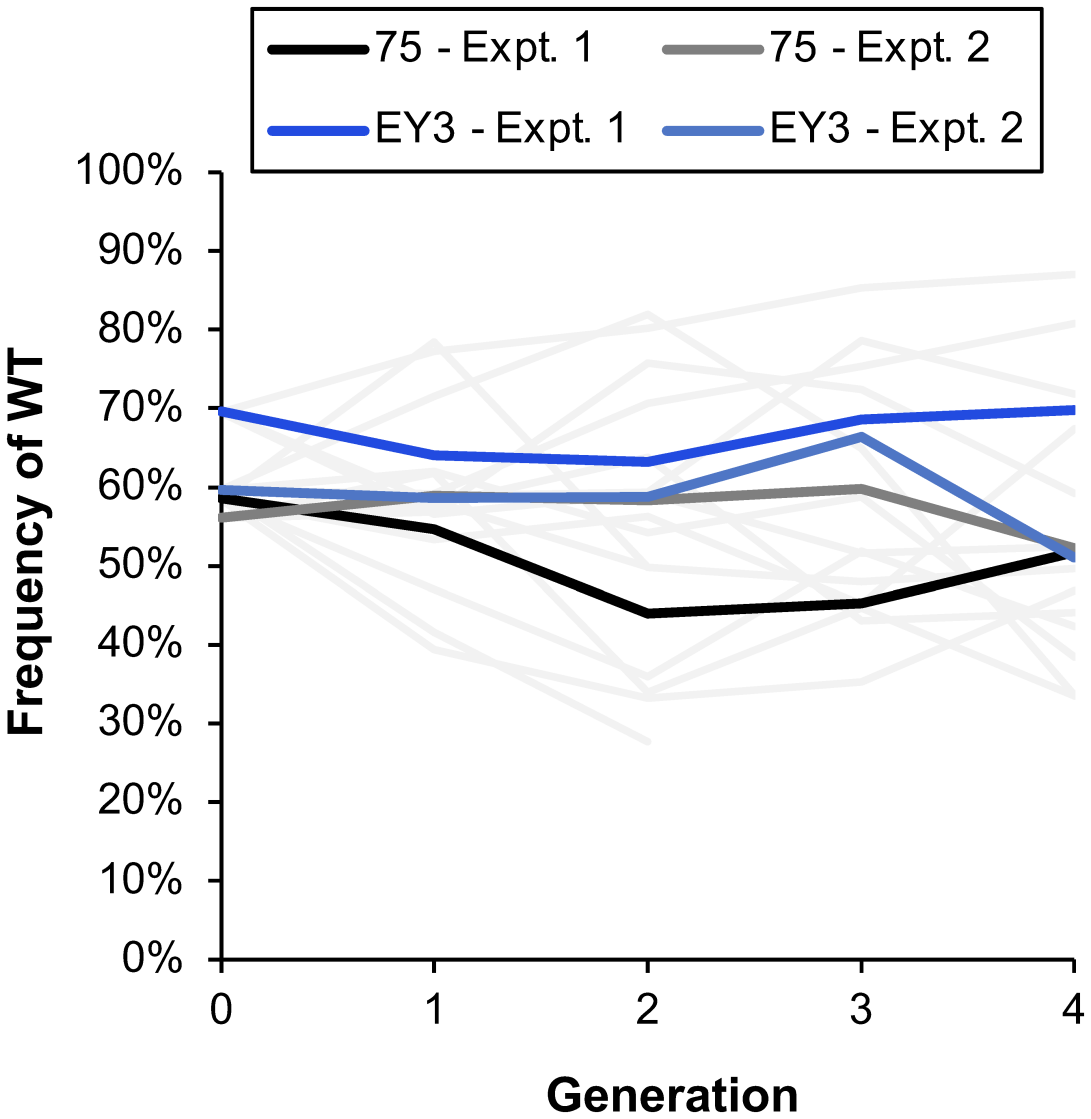
The estimated frequency of wild-type (WT) DNA quantified with a TaqMan qPCR assay that delineated between WT and mutant (MT) *Colletotrichum siamense* DNA. The average estimated frequency over the course of multiple generations of strains 75 and EY12-3 is a darker line, while thinner gray lines represent the different replicate treatments of each strain in the two independent experiments.

A multiplex TaqMan qPCR assay was developed to assess the ratio of WT : MT *C. siamense* DNA and was found to accurately quantify the WT : MT ratio of DNA extracted from known ratios of WT : MT spores (R^2^ > 0.9; **Supplementary Figure 2**). Blueberries were initially inoculated with a 50:50 ratio of WT : MT spores (50% WT frequency; generation 0) and the estimated WT frequency averaged 61% by the qPCR assay (**Figure 5**). Of the DNA extracted from the following generations, the estimated WT frequency averaged 59%, 56%, 58%, and 55% from generations 1, 2, 3, and 4, respectively. The data of the two repeated experiments was combined for analysis and there was not a significant change in WT frequency over the course of the generations (p = 0.958) and there was not a significant difference in WT frequency between the two strains 75 and EY12-3 (p = 0.538; **Figure 5**). The blueberries were initially inoculated with MT and WT isolates of the strains 75, EY12-3, K833, RR19-1S, but strains K833 and RR19-1S and some replicates of the EY12-3 strain did not cause enough blueberry disease to continue the experiment for multiple generations.

## Discussion

In this study, we have utilized an *in-vitro* assembled RNP complex to establish a HDR mediated CRISPR/cas9 gene-editing system for *C. siamense*. This marker free CRISPR/cas9 system was used to create the point mutation E198A in *C. siamense* isolates collected from 3 different plant hosts to study fitness of resistant isolates with identical genetic backgrounds. The CRISPR/Cas9 system has been developed for many filamentous fungal spp. but only one marker-free CRISPR/Cas9 system using *Colletotrichum sansevieriae* has been reported so far (Nødvig et al., 2015; Nakamura et al., 2019; Leisen et al., 2020). However, to our knowledge this will be the first study when plasmid free CRISPR/Cas9 system is utilized to create a point mutation in *C. siamense*. Our objective was to establish a plasmid-free RNP mediated CRISPR/Cas9 system that could be utilized for other *Colletotrichum* spp. In contrast to previously published methods that used Cas9 and sgRNA-encoding plasmids which were time consuming and less efficient, we used chemically synthesized Cas9 protein and RNA oligos to create a RNP complex (Arazoe et al., 2015; Foster et al., 2018). This study corroborated that RNP-mediated CRISPR/Cas9 editing is highly efficient, accurate, and quick, which has been demonstrated in a study conducted for gene editing in fungal and animal cells (Vakulskas and Behlke, 2019; Yang et al., 2020). Furthermore, *in vitro* synthesized DNA donor ssODN reduced the time and cost required to make gene edits in fungi and generate many transformants.

The use of donor oligos to create the point mutation in the TUB2 gene was highly specific as no evidence of off-target mutations in any of the resistant mutants of *C. siamense* were observed. Therefore, this system for point mutation is an extremely valuable and adaptable addition to *Colletotrichum* spp. gene editing. This system used the HDR pathway to repair the breaks generated by Cas9 in the DNA sequence. The use of marker free CRISPR-Cas9–mediated HDR for gene editing in the fungal genome has great potential; however, its utility has been limited by low efficiency, which requires recruitment of a donor DNA template near the site of cleavage through base pairing (Ling et al., 2020). To address this, ssDNA oligos (ssODNs) with the desired nucleotide base for point mutation were used to increase the efficiency of HDR. Overall, a highly efficient CRISPR/Cas9 system to create point mutations *C. siamense* isolates was established.

No clear indication of fitness cost associated with resistance to thiophanate-methyl in *Colletotrichum* species has yet been reported (Vieira et al., 2017). Differences in fitness between MT and WT isolates may be very slight and environmental condition-dependent. Some studies showed that temperature may be a factor affecting fitness costs associated with E198A and/or other mutations conferring MBC resistance (Oakley and Morris, 1981; Hawkins and Fraaije, 2018). In contrast to prior studies which collected various isolates which were sensitive or resistant to a fungicide for investigating fitness costs, the MT and WT isolates from this study were genetically identical (to the best of our knowledge) aside from the E198A mutation. Also, the parental WT isolates were collected from different hosts, and may have genetically diverse backgrounds. We evaluated commonly tested fitness metrics, such as mycelial growth rate, spore production, germination rate, infection severity, and infection incidence which reflects virulence of the isolates in their ability to reproduce, spread, and cause infections. Interestingly, mycelial growth rate, spore production, and blueberry incidence tended to be higher in mutant isolates than wild-type isolates, indications of increased virulence (**Figures 2A, B**, **4B**). On the other hand, germination rate tended to be lower in mutant isolates, indicating a fitness cost (**Figure 2C**). In the end, the increased virulence of mycelial growth rate and spore production may cancel out the decreased virulence of the lower germination rate associated with the E198A mutation in this study.

We also investigated if these MT and WT strains change in their fitness over the course of successive generations on artificial media. Prior studies have noted decreased virulence of plant pathogens after successive generations in the absence of the host (Breen et al., 2022). Interestingly, major decreases in virulence or changes between the MT and WT respective levels of fitness did not appear to occur over the course of 5 generations (**Figure 3**). Lastly, we evaluated the sensitivity to fungicides within the DMI and SDHI groups which are considered to be unrelated to MBC fungicides, because less fit isolates may be less tolerant to other stressors, such as fungicides of other chemical classes. Despite this, MT isolates were more penthiopyrad tolerant than WT isolates.

The direct competition between WT and MT isolates in the absence of fungicide selection pressure was evaluated through four inoculations of blueberries with WT/MT spore mixtures. Chen et al. (2016) documented a decrease in multifungicide resistance in *B. cinerea* resistance over three generations in a similar experiment, which may indicate a stronger cost to the competitive ability with regards to resistance to multiple fungicides in *B. cinerea*, than MBC resistance in *C. siamense.* Although a difference in competitivity was not observed after four generations with *C. siamense*, it is possible that a more significant shift in the WT : MT ratio could have occurred if the experiment was carried forward for many more generations or if more stressful conditions were created for the inocula.

Over all 41 fitness analyses in this study, WT isolates were significantly more fit than MT isolates in 7 analyses, MT isolates were significantly more fit than WT isolates in 10 analyses, and no difference between WT and MT isolates was observed in 24 analyses. Due to this, the MT isolates were as fit, if not more fit than the WT isolates. Studies of other fungi have also found an increase in virulence metrics associated with MBC-resistant isolates (Keinath and Zitter, 1998; Romero and Sutton, 1998; Baraldi et al., 2003). In many of our tests, there were no differences between the isolate types, yet a limited number of generations were evaluated. Further, some of the conditions that were investigated in this study involved colonies on artificial media and did not reflect the true conditions experienced by isolates in the field, such as seasonal, and diurnal conditions. The non-wounded blueberry inoculation experiments (MT caused higher disease incidence than WT) and the multigenerational competition experiments on non-wounded blueberries (no difference between MT and WT) were two tests in this study that contained more real-world conditions. More investigations of fitness under near real-world conditions, such as in live plants, and over longer timelines may reveal more differences between MT and WT populations. If the results of the tests in this study were to fully capture the severity of fitness costs associated with MBC resistance in *C. siamense*, the MBC resistance frequency in *C. siamense* populations would not decrease in the absence of usage of MBC fungicides.

In summary, an efficient CRISPR/Cas9 RNP-mediated genome editing system for *C. siamense* to develop MBC-resistant mutants containing the E198A mutation using HDR was generated in this study. This established system will facilitate more genome editing methods in other *Colletotrichum* spp. to create mutations by introducing *in vitro* assembled RNP which may avert the side-effects caused by traditional transgenic procedures using plasmids. Fitness and competitive ability of MT and WT isolates were investigated. This study demonstrated the utility of utilizing marker-free gene editing methods to evaluate fitness-costs associated with fungicide resistance. This may provide a clearer picture of the effects of resistance on fitness components than traditional methods that compare groups of isolates with different genetic backgrounds (Vieira et al., 2017).

## Data availability statement

Original datasets are available in a publicly accessible repository: The original contributions presented in the study are publicly available. This data can be found here: https://github.com/smallfruitUMD/Colletotrichum_siamense_fitness_CRISPR_2023.

## Author contributions

SC: Data curation, Formal Analysis, Investigation, Methodology, Software, Validation, Visualization, Writing – original draft. CA: Data curation, Investigation, Methodology, Writing – original draft, Writing – review & editing. MH: Conceptualization, Funding acquisition, Methodology, Project administration, Resources, Supervision, Writing – review & editing.

## Supplementary material

Supplementary Figure 1 Standard curves from serial dilutions of **(A)** wild-type (WT) or **(B)** mutant (MT) *Colletotrichum siamense* DNA from a TaqMan qPCR assay.

Supplementary Figure 2 The calculated frequency of wild-type (WT) spores from three separate runs of a TaqMan qPCR assay with wild-type (WT) and mutant (MT) DNA-specific probes with DNA extracted from known frequencies of WT spores.
