# Supplementary figures and images for: CRISPR-enabled investigation of fitness costs associated with the E198A mutation in β-tubulin of Colletotrichum siamense
Source: Front Plant Sci. 2023 Nov 3;14:1278133. doi: 10.3389/fpls.2023.1278133 (PMC10654983; doi:10.3389/fpls.2023.1278133)

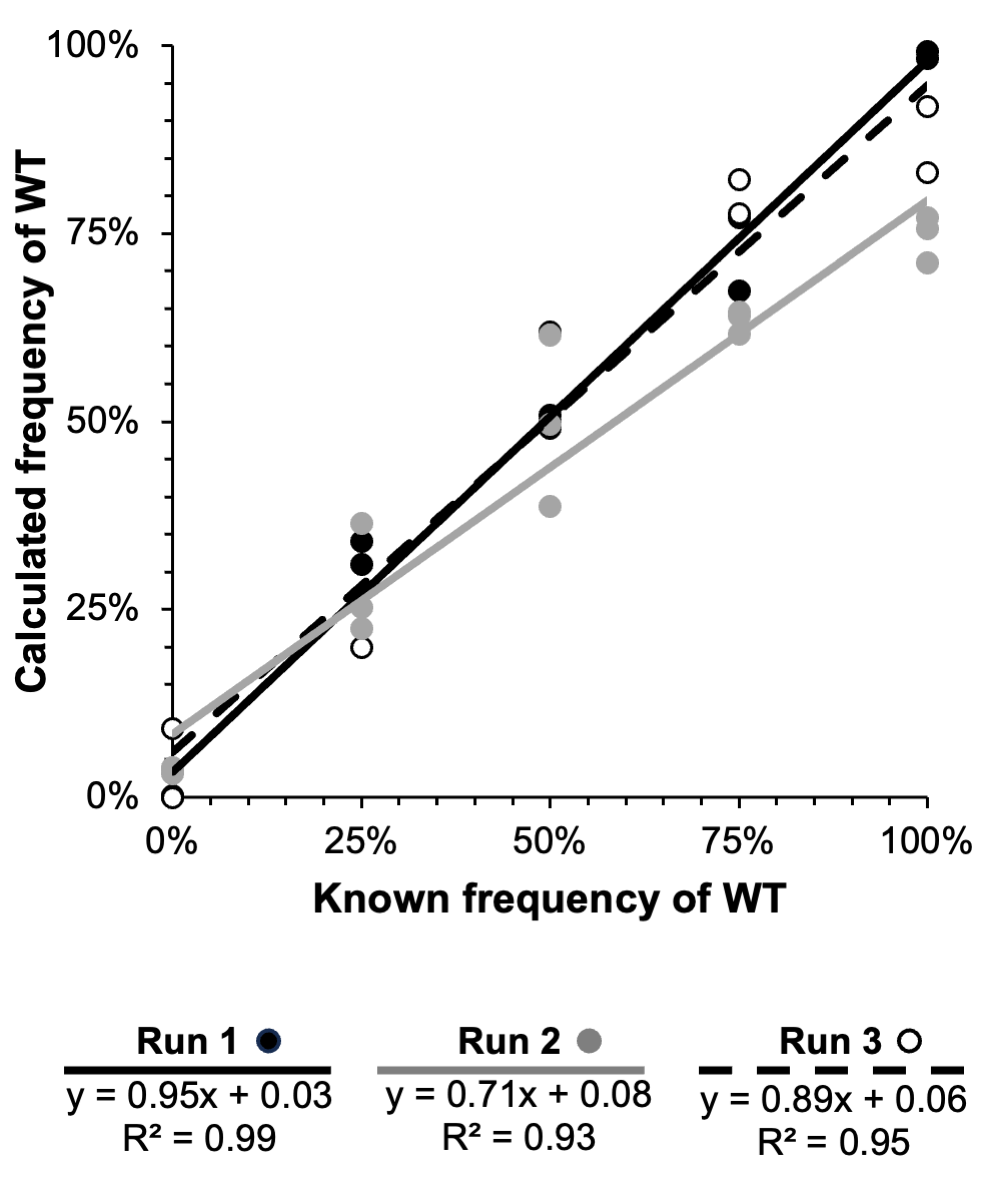

Supplement: Supplementary Figure 2 — The calculated frequency of wild-type (WT) spores from three separate runs of a TaqMan qPCR assay with wild-type (WT) and mutant (MT) DNA-specific probes with DNA extracted from known frequencies of WT spores. [file Image_2.tiff]
